# Hierarchical annotation of eQTLs by H-eQTL enables identification of genes with cell type-divergent regulation

**DOI:** 10.1186/s13059-024-03440-2

**Published:** 2024-11-25

**Authors:** Pawel F. Przytycki, Katherine S. Pollard

**Affiliations:** 1 Gladstone Institutes, San Francisco, CA USA; 2 Chan Zuckerberg Biohub, San Francisco, CA USA; 3 Department of Epidemiology and Biostatistics, Institute for Computational Health Sciences, Institute for Human Genetics and University of California, San Francisco, CA USA; 4 Present address: Faculty of Computing & Data Sciences, Boston University, Boston University, Boston, MA USA

## Abstract

While context-type-specific regulation of genes is largely determined by cis-regulatory regions, attempts to identify cell type-specific eQTLs are complicated by the nested nature of cell types. We present hierarchical eQTL (H-eQTL), a network-based model for hierarchical annotation of bulk-derived eQTLs to levels of a cell type tree using single-cell chromatin accessibility data and no clustering of cells into discrete cell types. Using our model, we annotate bulk-derived eQTLs from the developing brain with high specificity to levels of a cell type hierarchy, which allows sensitive detection of genes with multiple distinct non-coding elements regulating their expression in different cell types.

**Supplementary Information:**

The online version contains supplementary material available at 10.1186/s13059-024-03440-2.

## Background

Context-specific regulation of gene expression is largely determined by noncoding cis-regulatory regions [1, 2]. These sequences encode information about the time, place, and quantity in which a gene will be transcribed allowing for tissue and cell type-specific regulation [1, 2]. While it is well established that genes are pleiotropic [1, 2], the way in which regulatory elements specify the contexts in which a gene will be expressed is complex and not well understood [2].

An expression quantitative trait locus (eQTL) is a statistically significant association between a genetic variant (eSNP) and the expression of a gene (eGene), typically measured across large cohorts. In order to capture changes in expression in specific tissues, large-scale efforts such as GTEx take expression measurements in tissues across many individuals [3]. However, because many regulatory effects are cell type-specific, recent work has begun to identify cell type-specific eQTLs by taking massive amounts of single-cell gene expression measurements (scRNA-seq) across cohorts [4–6]. Unfortunately, such approaches are not broadly feasible due to the high cost of single-cell sequencing [6]. An alternative approach is to deconvolve bulk derived eQTLs into cell type-specific signatures based on scRNA-seq data for those cell types [7] or based on single-cell chromatin accessibility of the region containing the genetic variant [8, 9].

Attempts to identify cell type-specific eQTLs are complicated by the nested relationships between cell types. While most analyses assume discrete cell types [10], complex tissues such as the brain contain many rare and highly correlated cell types and subtypes that are not entirely distinct from each other [11, 12]. Cell states further complicate discretization. Therefore, the typical approach used with single-cell sequencing data of first generating clusters of similar cells and then assigning cell type labels to whole clusters often fails to capture the true diversity of cell types [11]. This in turn constrains the identification of the context in which genes are expressed from single-cell sequencing data to the most common and most distinct cell types.

Towards addressing these challenges, we present hierarchical eQTL (H-eQTL), a network-based model for hierarchical annotation of bulk-derived eQTLs using single-cell chromatin accessibility data (scATAC-seq). Our model explicitly takes into consideration the tree-based organizational principle underlying cell diversity [13], rather than treating cell type as a categorical variable, and scores bulk eQTLs at all levels of a cell hierarchy to best identify significant cell type and subtype-specific annotations. These scores are based on chromatin accessibility of the eSNP for each eQTL across cell types. Because our model annotates the genetic variant rather than the associated eGene, we allow for gene pleiotropy and associate each variant in a locus with the expression of the target eGene in potentially unique cell types.

We applied H-eQTL to eQTLs from the developing human brain [9], a complex organ with many rare or nested cell types and subtypes [14, 15]. We annotated 5889 of these bulk-derived eQTLs with high specificity to levels of a cell type hierarchy. Based on these hierarchical cell type labels, we identified 613 eGenes with multiple eSNPs that are specific to distinct cell types that we term cell type-divergent eQTLs. Using multiome single-cell accessibility and expression data [16], we confirmed that cells in which the given eSNP is accessible express the linked eGene in the multiple predicted contexts. Finally, we dissected the regulation of *FABP7* and *ICA1L*, two genes expressed in the developing brain with multiple cell type-divergent eQTLs, using Massively Parallel Reporter Assay (MPRA) data [17]. We observed that both genes have eSNPs with independent regulatory effects in the developing brain, confirming that they are functional variants. Overall, our hierarchical method generated an annotation of bulk eQTL data that allowed for the discovery of divergent cell type regulation in an organ with a complex mixture of cell types.

## Results

### Hierarchical model for nested cell types

**Fig. 1:**
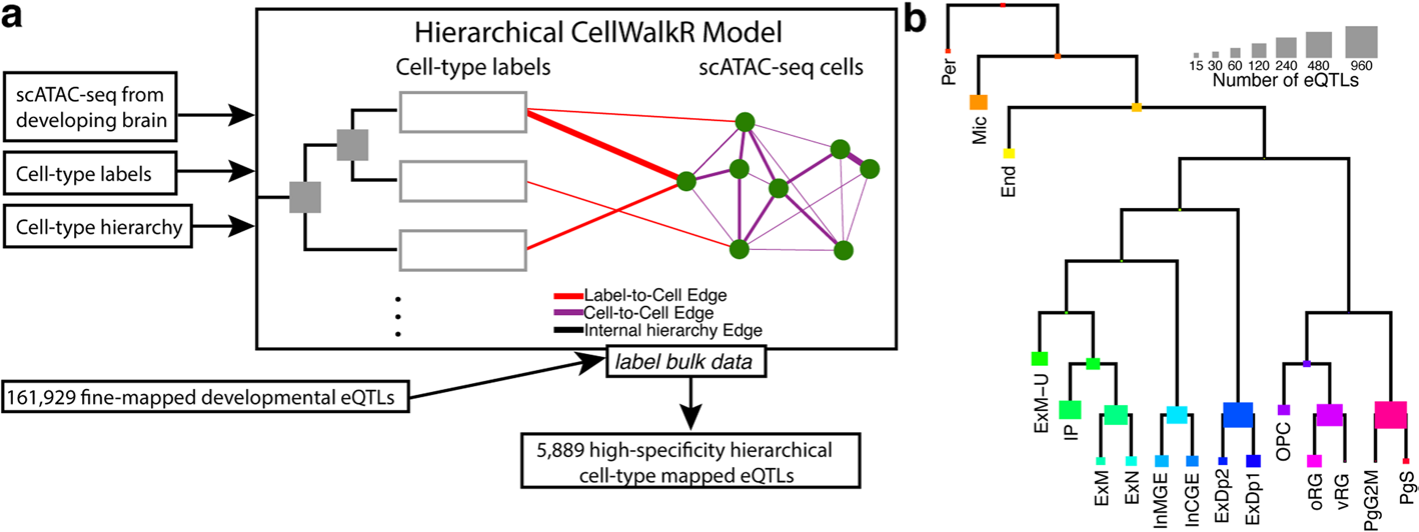
Hierarchical cell type mapping by H-eQTL. **a** The H-eQTL workflow: the hierarchical model extends CellWalkR to take a cell type hierarchy as an input in addition to scATAC-seq data and cell type labels. The hierarchy is used to create internal nodes in the model that correspond to cell types higher in the hierarchy. This hierarchical model was used to label a large set of fine-mapped developmental brain eQTLs with high specificity. **b** Count of how many developmental brain eQTLs are mapped to each hierarchical cell type

We developed H-eQTL, a network-based hierarchical model to identify cell type-specific eQTLs in complex tissues with closely related and nested cell types (Fig. 1a). Our model extends the existing CellWalkR model [18] to take a cell type hierarchy as input in addition to cell type labels and scATAC-seq data. Briefly, the cell type hierarchy is taken as prior knowledge, and it is implemented as edges between leaf nodes that represent specific cell types and internal nodes that represent broader cell types higher in the hierarchy. The cell type nodes are then connected to nodes representing cells based on how well marker genes correspond to each cell’s chromatin accessibility, and cells are connected to each other based on the similarity of their genome-wide chromatin accessibility. A random walk with random restarts model of network diffusion is then run on this network to calculate how much information flows from each node to each other node. In particular, this includes the probability that a walk starting at each cell node ends at each cell type node as well as each internal node representing portions of the cell type hierarchy.

Next, given a set of bulk-derived eQTLs, each eQTL is mapped to hierarchical cell types using the calculated flow of information and the chromatin accessibility of individual cells. For each eQTL, the location of the eSNP is intersected with the accessibility of each cell, and then the normalized cumulative flow from those cells is used to score each hierarchical cell type. This results in a label *z*-score for each hierarchical cell type for each eSNP. In this way, eQTLs from bulk data can be mapped to a cell type tree. The cell type with the highest score can be used as a discrete labeling of the eQTL, or the scores across all cell types can be treated as a fuzzy (i.e., probabilistic) labeling.

### Annotation of eQTLs in the developing brain

We next applied this hierarchical model to label bulk eQTLs from the developing brain [9] using scATAC-seq data from the mid-gestation telencephalon [19] combined with a transcriptomics-based cell type hierarchy derived from similar samples [15]. Our model was able to label 5889 eQTLs to hierarchical cell types with high specificity (*z*-score greater than 2, see the “Methods” section for details). These eQTLs mapped to a large variety of hierarchical cell types (Fig. 1b, see Additional file 2: Table S1 for full names of cell types), including both specific cell types (e.g., outer vs ventral radial glia) as well as higher level annotations (e.g., broadly neuronal). A full list of cell type annotations for eQTLs is provided in Additional file 3: Table S2.

**Fig. 2:**
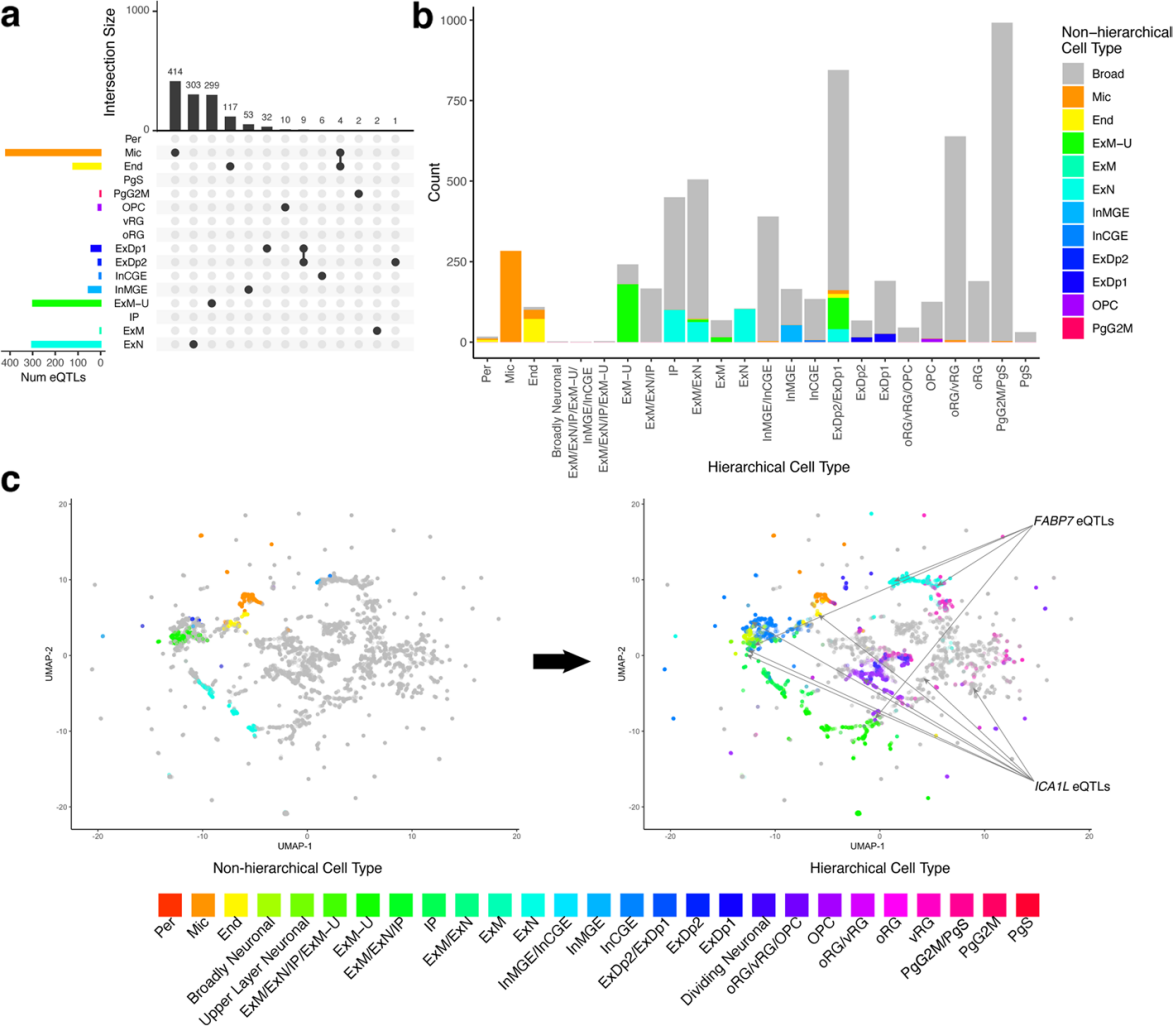
Hierarchical cell types provide improved labeling. **a** Non-hierarchical high-specificity cell type mapped eQTLs generally map to a single cell type, but they are biased towards very distinct or common cell types. **b** Many eQTLs that could not be mapped to a specific cell type in the non-hierarchical model (“Broad,” shown in gray) receive hierarchical cell type labels (shown on *x*-axis). **c** UMAP embedding of eQTLs labeled by non-hierarchical cell type (left) and hierarchical cell type (right) shows that a diverse set of previously unlabeled eQTLs can now be labeled. Due to this increased label diversity, we can observe that some eQTLs for the same gene (e.g., *FABP7* and *ICA1L*) map to vastly different cell types

For comparison, we annotated eQTLs with a non-hierarchical version of the same model. We found that without hierarchical cell types, while the model was still able to label highly distinct cell types such as endothelial cells and microglia, it was unable to label similar or nested cell types such as different radial glia (Fig. 2a, Additional file 1: Fig. S1a and b). Only 1252 eQTLs could be mapped to non-hierarchical cell types with high specificity, indicating a fivefold loss in annotation compared to using the cell type tree (Fig. 2b). Of those that could be annotated by the non-hierarchical model, 81.4% received the same annotation from the hierarchical model, within a level of the hierarchy (direct parents or siblings). A less stringent threshold for specificity (*z*-score greater than 1) annotates more eQTLs but maps multiple non-hierarchical cell types to each eQTL (Additional file 1: Fig. S1c), likely due to related cell types having highly correlated scores (Additional file 1: Fig. S2). This indicates that a major advantage of using a cell type tree is its ability to account for highly correlated cell types.

As an orthogonal comparison, we overlapped eSNPs with annotated broad cell type-specific peaks and enhancers [19] and compared these annotations to our hierarchical and non-hierarchical eQTL cell type labels. Overall, we observed that our labels are consistent with these two sources of regulatory element annotation (Additional file 1: Fig. S3). For example, we found that 93% of our hierarchically labeled eQTLs have eSNPs that overlap cell type-specific peaks. Non-hierarchical eQTL labels were also generally consistent with the annotations, but fewer of them overlapped cell type-specific peaks and enhancers compared to hierarchical labeling. Together, these analyses validate our cell type labels and underscore the extra sensitivity provided by the cell type tree.

Using the label scores we calculated for each hierarchical cell type for each eQTL, we embedded the eQTLs into two-dimensional UMAP space (Fig. 2c). Consistent with the previous results, we observed a large increase in the coverage and diversity of hierarchical annotations of eQTLs as compared to non-hierarchical annotations. Hierarchically related cell types are located near each other, reflecting their relationships being modeled in the eQTL labeling process. Furthermore, eQTLs tend to cluster by cell type, rather than by the eGene each eSNP is linked to (*p*-value < 0.05 using a two-tailed Wilcoxon rank-sum test for distances between pairs of eSNPs assigned to the same gene versus to the same hierarchical cell type). For example, the four eQTLs for the gene *FABP7* are annotated to three different hierarchical cell types, and the six eQTLs for *ICA1L* are annotated to four different hierarchical cell types. These multi-cell type annotations were not detected with the non-hierarchical model or by overlapping with cell type-specific peaks, emphasizing the need for hierarchical cell type annotation.

### Identification of cell type-divergent eQTLs

**Fig. 3:**
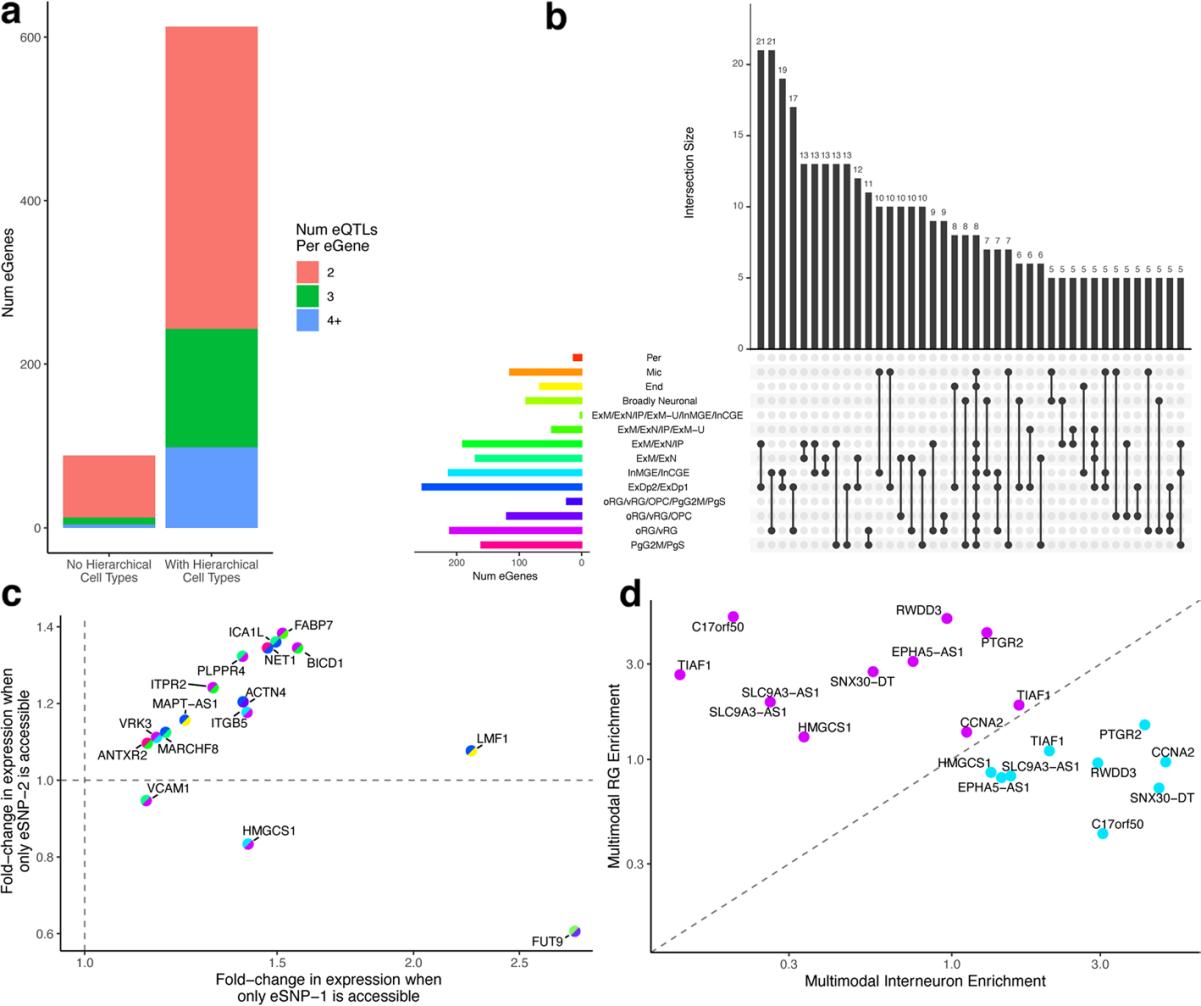
Cell type-divergent eQTLs. **a** While only 66 eGenes have at least two eQTLs with distinct non-hierarchical cell types, the larger number of annotations we can make with the hierarchical model results in 613 eGenes having at least two distinct hierarchical cell types. **b** An upset plot showing the most common divergent hierarchical cell types for eQTLs across eGenes. **c** For highly expressed genes with cell type-divergent eQTLs, the gene is differentially expressed in cells where the first eSNP is accessible (*x*-axis) as well as in cells where the second eSNP is accessible (*y*-axis), as observed in jointly profiled multiome scRNA/ATAC data. The split colors of each point indicate the divergent hierarchical cell types following the key from panel b. **d** For highly expressed genes with divergent radial glia (oRG/vRG, shown in magenta) and interneuron (InMGE/InCGE, shown in teal) eQTLs, cells in which the respective eSNP is accessible are enriched for the matching cell type in the labeled scRNA-seq portion of jointly profiled multiome scRNA/ATAC data

Given the hierarchical model’s increased ability to assign multiple distinct cell types to different eSNPs linked to the same eGene, we sought to identify all such genes. For each eGene, we considered it to have cell type-divergent eQTLs if at least two eSNPs linked to that eGene were not the same cell type nor were they ancestors of each other in the original cell type hierarchy. We also required that the full label scores for the eSNPs were not similar to each other (see the “Methods” section for details). We identified 613 eGenes with cell type-divergent eQTLs, the majority of which were linked to two distinct cell types though a few had three or more cell type annotations (Fig. 3a). For comparison, only 88 eGenes with cell type-divergent eQTLs could be identified using the non-hierarchical model, only 320 using any overlaps with annotated cell type-specific peaks, and only 33 using overlaps with cell type-specific enhancers (Additional file 1: Fig. S4). Thus, the higher sensitivity of our hierarchical model revealed a greater frequency of genes with eSNPs that function in distinct cell types.

Taking a closer look at eGenes with cell type-divergent eQTLs, we find eGenes with eSNPs corresponding to diverse combinations of cell types such as deep layer plus maturing excitatory neurons (21 eGenes) and interneurons plus radial glia (21 eGenes) (Fig. 3b). For comparison, the non-hierarchical model almost exclusively identified cell type-divergent eGenes with eSNPs involving microglia and endothelial cells, two particularly distinct cell types, and generally failed to identify divergent eQTLs annotated to different types of neurons (Additional file 1: Fig. S5). Additionally, we observed that the more cell type-specific eSNPs an eGene has, the higher its expression entropy across cell types in scRNA-seq [16] (Additional file 1: Fig. S6). This indicates that the more different cell type-specific eSNPs an eGene has, the more diverse the expression of that eGene across cell types, consistent with these eQTLs providing cell type-specific regulation and making the gene more pleiotropic.

### Genes with cell type-divergent eQTLs exhibit cell type-specific regulation

In order to determine if cell type-divergent eQTLs directly contribute to cell type-specific expression, we looked at multiome measurements of scRNA-seq and scATAC-seq in the same cells in the developing brain [16]. Given the sparse nature of multiome data, only 356 eGenes with cell type-divergent eQTLs could be tested in the multiome data. For each of these eGenes and each of their eSNPs, we tested whether cells in which the eSNP was uniquely accessible (i.e., no other eSNP was accessible for that eGene) the eGene was differentially expressed relative to cells in which the eSNP was not accessible. We observed significant differential expression across multiple eQTLs for 30 of these eGenes (false discovery rate < 0.05, Additional file 1: Fig. S7 and S8). Sixteen of these differentially expressed eGenes were highly expressed (Fig. 3c). Since accessibility and expression were determined using very sparse data per cell, we posit that the lack of significant differential expression for most eQTLs is influenced by low power. Overall, we observe that changes in accessibility in cell type-divergent eQTLs lead to changes in expression in those same cells.

Next, we considered the previously determined cell type annotations of the scRNA-seq portion of the multiome data in order to determine if predicted cell type-divergent eQTLs had eSNPs with cell type-specific accessibility. While these multiome cell type annotations do not directly match the labels used in our cell type hierarchy, we observe that generally they are enriched in the predicted cell type when the corresponding eSNP is accessible (Additional file 1: Fig. S9). Furthermore, for highly expressed eGenes with cell type-divergent eQTLs, we detected an enrichment for corresponding cell types when each eSNP was uniquely accessible (Additional file 1: Fig. S10). For example, looking at eGenes that have divergent eQTLs for radial glia and interneurons, we see that the eSNP is always more enriched in the predicted cell type when the corresponding variant is accessible (Fig. 3d).

Finally, we looked for mechanisms of action for the change in expression. We found that of the 613 eGenes with cell type-divergent eQTLs, 222 had eSNPs that disrupted binding sites of at least two different transcription factors (TFs) that are expressed in the corresponding cell type. Among these TFs, some are very specific to a single hierarchical cell type (e.g., *FOSB* and *JUND* for cycling PgG2M/PgS progenitor cells, *FOXP2* for newborn and maturing ExM/ExN/IP excitatory neurons), while some are disrupted in many cell types (Additional file 1: Fig. S11). Furthermore, some TFs frequently co-occur as disrupted by cell type-divergent eQTLs (Additional file 1: Fig. S12). For example, 13 genes have both an eSNP predicted to disrupt *SMAD2* binding in newborn and maturing excitatory neurons (ExM/ExN/IP) and an eSNP predicted to disrupt *JUND* binding in cycling progenitor cells. This supports the idea that one mechanism of gene pleiotropy is cell type-specific transcription factor binding.

### Cell type-divergent regulation of FABP7 and ICA1L

**Fig. 4:**
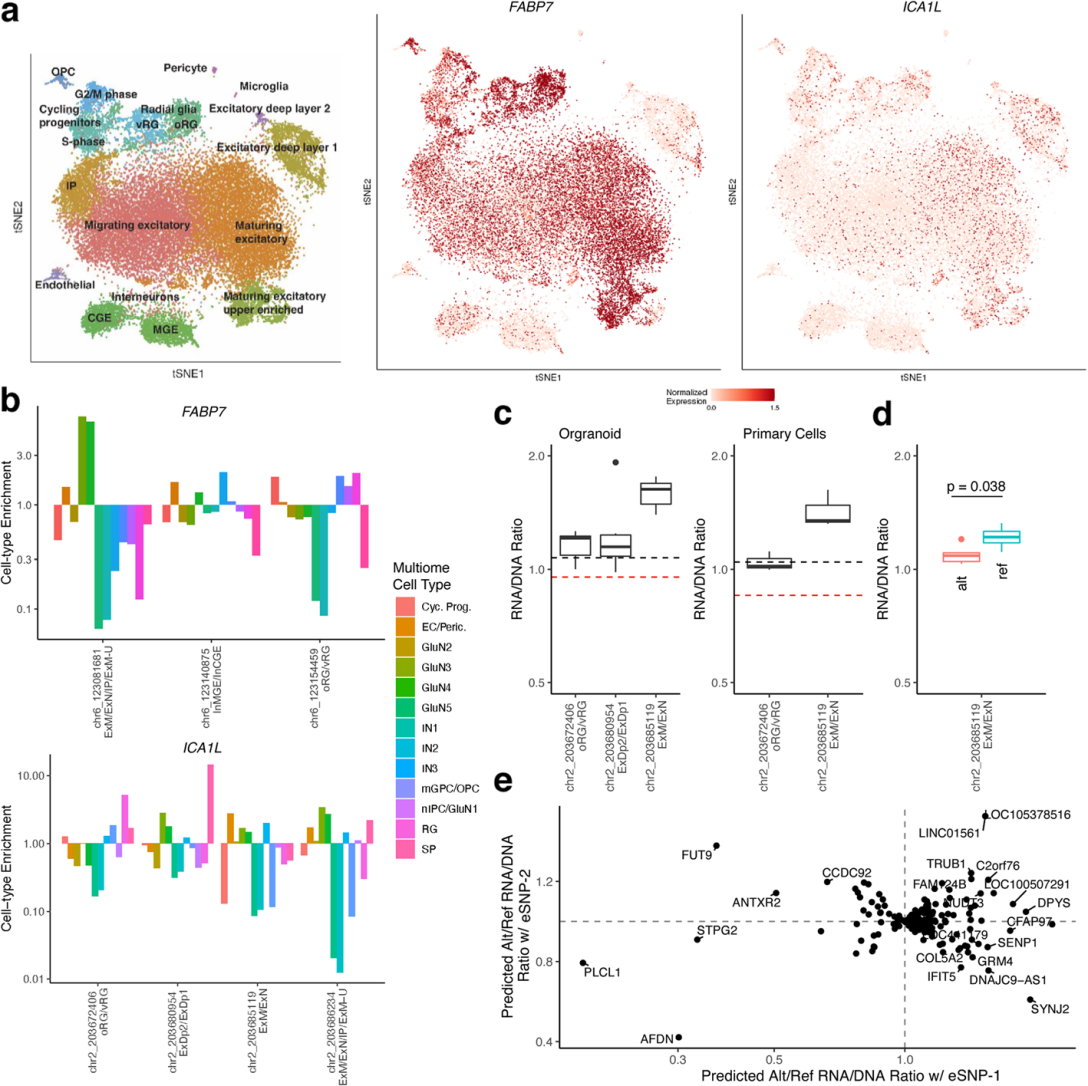
*FABP7* and *ICA1L* exhibit cell type-specific regulation. **a** *FABP7* (middle) and *ICA1L* (right) are expressed in many cell types in the developing brain (cell type labels shown on left). **b** For each eQTL associated with *FABP7* (top) and *ICA1L* (bottom) cells in which the respective eSNP is accessible are enriched for the matching cell type in the labeled scRNA-seq portion of jointly profiled multiome scRNA/ATAC data. **c** MPRA data from brain organoid (left) and primary cells (right) that overlapped *ICA1L* eSNPs all showed RNA/DNA ratio at or above the median of positive controls (black dashed line) and above the median for negative controls (red dashed line) indicating that these variants occur in functional regulatory regions. **d** For one *ICA1L* eSNP, both the reference (red) and alternative (blue) alleles were tested in an MPRA finding a significant difference in RNA/DNA ratio. **e** A machine learning model predicts that the ratio between alternative and reference alleles of RNA/DNA ratio for genes with cell type-divergent eQTLs is often independently changed by both eSNPs

The brain-related genes *FABP7* and *ICA1L* are both expressed in multiple cell types in the developing brain (Fig. 4a). *FABP7*, which plays a role in the establishment of radial glial fiber [20], has four eQTLs that we mapped to hierarchical cell types. Of the three eSNPs that overlapped peaks in multiome scRNA/ATAC data, each was enriched for the corresponding scRNA-seq cell type annotation when the variant was accessible (Fig. 4b, top). Furthermore, two eSNPs overlapped known enhancers, two overlapped predicted cell type-specific regulatory elements, and three disrupted different TFs that were expressed in the corresponding cell types, indicating a possible mechanism of action (Additional file 1: Fig. S13). Taken together, this suggests that the expression of *FABP7* in different cell types may be driven by cis regulatory elements overlapping eSNPs as annotated by our hierarchical model.

A similar enrichment for corresponding cell types was observed for *ICA1L* eQTLs, four of which could be tested using multiome data (Fig. 4b, bottom). For three of these eSNPs, the variant site in question had previously been tested in an MPRA experiment conducted in cortical organoids and primary cortical cells [17]. In all three, the MPRA activity (RNA/DNA ratio) was greater than or equal to positive controls, and always higher than negative controls (Fig. 4c), indicating that these are functional variants. Furthermore, for one variant, both the reference and alternative allele were tested in the MPRA, and there was a significant difference in the RNA/DNA ratio between alleles (Fig. 4d). This data validates our prediction of cell type-specific regulatory variants for *ICA1L.*

In order to estimate the impact of the change from the reference allele to the variant allele for each eQTL, we expanded our analysis using a machine learning model trained on MPRA data [17]. While this model is not able to make cell type-specific predictions, it helps determine if a specific eQTL may be functional. We predicted RNA/DNA ratios for both the reference allele and alternative allele for the eSNP for each eQTL and found that among eGenes with cell type-divergent eQTLs, 23% had an eSNP that was predicted to be functional (defined as an absolute log-fold change in the ratio between alleles greater than 0.2, the threshold for significance at a false discovery rate < 0.1 in the original MPRA study). Of these, 16% had a second eSNP that was annotated to a different hierarchical cell type and was also predicted to be functional (Fig. 4e). Thus, machine learning provides further support for the presence of many eGenes with cell type-divergent eQTLs regulating different components of their expression across the developing brain.

## Discussion

Hierarchical cell type annotation by H-eQTL enabled the identification of eQTLs with cell type-divergent regulation of genes. Crucially, it showed clear advantages over using a non-hierarchical approach or simply overlapping the eSNPs of eQTLs with annotated enhancers or regulatory regions. Additionally, our approach gives a continuous score to each eQTL across every level of the cell type tree rather than just a binary annotation for each cell type. By not selecting a particular resolution of cell type and not relying on any prior clustering of cells, our approach allows for a more flexible view of annotation. It also leverages relationships between cell types to annotate eQTLs.

Our observation that changes in accessibility around eSNPs link to cell type-specific expression of genes, potentially driven by altered transcription factor binding motifs, agrees with the currently held views of gene pleiotropy. However, lingering questions on the combinatorial operation of enhancers and pleiotropic enhancers were not addressed in this study. Furthermore, we are not able to capture the phenomenon of enhancer priming in which transcription factors may bind enhancers without directly affecting gene expression but rather facilitate activation by subsequent, more specific, transcription factors [21]. This has been observed in neural progenitors [22]. The notion that this effect could take place in cell type hierarchies with accessibility and gene expression manifesting on different levels of the hierarchy is an intriguing motivation for future investigations. With a larger and higher read depth set of single-cell sequencing data it would be feasible to use our same approach to study such phenomena. Unfortunately, current scATAC-seq coverage is too low.

While we aimed to detect cell type-specific effects of eQTLs, there are important caveats to our ability to identify these. First, there are major limitations to working with bulk-derived eQTLs. While the goal of the method developed in our manuscript is to annotate such eQTLs, we expect that many cell type-specific eQTLs are simply missed due to low prevalence in bulk samples. However, our method could also be applied to cell type-specific eQTLs to annotate them using cell type labels from an external source (including mapping them to more specific cell states) or to get a probabilistic mapping for labels from the same dataset. Furthermore, the vast majority of the eQTLs we set out to study are unlikely to be causal [9]. While overlapping eSNPs with chromatin accessibility, transcription factor binding sites, and functional regions as observed with MPRAs enriches for functional variants, causality is more difficult to establish. In addition to this, these functional annotations were not able to capture cell type-specific effects. In the future, there is the potential to examine cell type-specific effects more directly through single-cell MPRAs [23] and experiments combining CRIPSPRi/a with gene expression read-outs in sorted cell types [24].

Another important caveat is the enormous variety in the availability and quality of cell type hierarchies. Detailed hierarchies are the results of many years of research and have only recently started to become more abundant. Examples include a cell type tree of prenatal mouse development [25], *C. elegans* embryogenesis [26], hematopoiesis [27], and *D. melanogaster* embryogenesis [28]. On the other end, basic hierarchies can be generated directly from scRNA-seq data with computational methods such as treeArches [29] and CellHint [30]. Notably, our proposed method also works with partial hierarchies in cases where there is a limited amount of prior knowledge. Beyond cell type hierarchies, there is also the caveat that the continuity of cell states cannot be captured by a tree. Our model is bound to miss subtle shifts in cells, such as those that may occur during transitions in development or disease.

The hierarchical model we propose can be readily extended to more complex representations of cell types beyond cell type trees. While we directly encode cell type trees as parent nodes each with two child nodes, our framework allows for the cell types to be any graph. This includes more than two descendant cell types for each parent as well as cell types that are ambiguously descended from multiple parents. Furthermore, edges can be weighted to represent the probability of two cell types being related or descended from each other. Overall, a graph provides a flexible model of cell type relationships.

Finally, while this study focused on eQTLs with the goal of examining gene pleiotropy, hierarchical annotation can be applied more generally. Our model could be used to hierarchically annotate any genomic regions, including bulk-derived regulatory elements, GWAS hits or other noncoding variants, and more. While each of these different applications would require a careful study of the corresponding data to build the correct network representation, the general framework we have proposed is universal.

## Conclusions

In summary, we have shown that a hierarchical representation of cell types allows for robust labeling of bulk-derived eQTLs with scATAC-seq data. This improved labeling in turn allowed us to identify genes with cell type-divergent regulation providing a promising avenue for studying gene pleiotropy.

## Methods

### Hierarchical model construction

The hierarchical H-eQTL model was implemented as an extension of the CellWalkR package (version 0.99.1) [18]. First, a cell type hierarchy, which is typically represented by a tree in which the leaf nodes are cell types, is converted into a symmetrical (2*n*−1)-by-(2*n*−1) adjacency matrix where *n* is the number of cell types in the tree, representing the total number of leaf and internal nodes in a tree. Each parent–child relationship in the tree is given a value of one in the adjacency matrix, and all other values are set to zero. Next, a (2*n*−1)-by-*c* matrix, where *c* is the number of cells, is constructed by padding the *n*-by-*c* label-cell matrix constructed by CellWalkR from cell type labels and scATAC-seq data with an (*n*−1)-by-*c* matrix of zeros, representing that no internal tree nodes are directly connected to cells. Finally, a symmetrical (2*n*−1 + *c*)-by-(2*n*−1 + *c*) matrix is constructed by appending the (2*n*-1)-by-(2*n*-1) matrix, the (2*n*−1)-by-*c* matrix, and transposed *c*-by-(2*n*−1) copy of that matrix and the *c*-by-*c* cell–cell matrix generated by CellWalkR to each other. The cell-to-cell and cell-to-label edges of the CellWalker graph in this study are computed in the same way as previously described [8, 18]. Briefly, the cell-to-cell edges are based on similarity of genome-wide chromatin accessibility profiles of each pair of cells. The label-to-cell edges are based on accessibility of cell type-specific marker genes, which can be identified using single-cell RNA-seq.

Once the network matrix incorporating hierarchical cell type relationships is constructed, it is used in place of the standard matrix generated by CellWalkR for all downstream functions. A random walk with random restarts as implemented in CellWalkR’s *randomWalk* function calculates how much information flows from each node to each other node. A walk can start on any cell or label, including internal nodes of the tree, and similarly can end on any cell, label, or internal node. CellWalkR can either solve for the convergence of this random walk directly as F = α(I − (1 − α)W)^−1^ or iteratively as F_t+1_ = αI + (1 − α)WF_t_, where W = D^−1^A, D is a diagonal matrix of the sums of edge weights for each node, and A is the adjacency matrix representing the graph. The influence matrix F gives the probability that a walk starting at each node in the graph will end at each other node in the graph (including internal nodes of the hierarchy). One can focus an analysis on the subset of entries in the influence matrix F corresponding to paths of interest. For the eQTL analyses in this manuscript, we only use the probabilities for walks started on cells and ended at a cell type node, including internal nodes of the cell type hierarchy.

Annotations, such as eSNPs or regulatory elements detected in bulk data, are mapped to each cell type node and each internal node using CellWalkR’s *labelBulk* function, which calculates the total probability that walks starting at cells in which the annotation (in this study, an eSNP) is accessible end at each label. After the stochastic steady-state influence matrix F is calculated, the locations of eSNPs are overlapped with chromatin accessibility of all individual cells. For each cell where the eSNP is accessible (i.e., where at least one scATAC-seq read overlaps the eSNP), we extract the probability that a walk from that cell ends at each node of the cell type tree. These probabilities are then summed across all the cells from which walks started. In other words, the overlap of scATAC-seq reads with a given eSNP is used to select which cells should be used, after which point the random walks are used to assign hierarchical cell types to the eSNPs. Thus, for a given eSNP, the cell type label is determined using the label probabilities for all cells in which the eSNP is accessible. This approach is designed to be robust to noise and sparsity in scATAC-seq data from individual cells by using genome-wide chromatin accessibility of cells (specifically accessibility of cell type marker genes) and summing over cells.

### Cell type eQTL scoring

To score eQTLs from the developing brain, we first built a hierarchical model for cell types in the developing brain. The cell type hierarchy from Polioudakis et al. [15] was encoded as described above with 16 leaf nodes (corresponding to cell types) and 15 internal nodes. We then ran H-eQTL using cell type marker genes from Polioudakis et al. and scATAC-seq from Ziffra et. al. [19] with the “logFC” option for marker genes corresponding to cell types and the *mapSnapATACToGenes* function with the “whichMat” option set to “gmat” to generate label-cell edges and *computeCellSim* to generate cell–cell edges. We tuned edge weights using the *tuneEdgeWeights* function with steps set to three and found that 100 was the optimal setting of the edge weight parameter. We ran the *walkCells* function with the optimal edge weight to calculate a final influence matrix.

161,929 fine-mapped eQTLs were taken from Wen et al. [9] and lifted from hg19 to hg38 using liftOver [31]. They were then mapped to each of the 16 cell types and 15 internal nodes using the *labelBulk* function. This resulted in a vector of 31 scores for 11,765 eSNPs across 4137 eGenes, corresponding to the set of eQTLs that could be scored based on the scATAC-seq data (meaning they overlap at least one read from at least one cell in the scATAC-seq data). Of these, 5889 mapped to at least one node with a label *z*-score greater than 2, indicating high specificity. Next, for any eQTL where a parent node and both child nodes had label *z*-scores greater than 2, only the parent label was kept, with this process iteratively applied from the leaf nodes up to the root of the tree. This is often the case for the parent to two highly correlated leaf nodes but is rarely the case higher up the tree where the nodes are not as correlated. For downstream analyses, for each eQTL, we then further checked to see if it was significant in more than one hierarchical cell type. As a conservative annotation approach, in cases where an eQTL was significant in cell types that were ancestors of each other in the hierarchy, we only considered the highest level (i.e., least specific) annotation, and removed any more specific annotations.

For comparison to a non-hierarchical annotation, we ran a standard version of CellWalkR with the same data and options and found that 100 was the optimal setting of the edge weight parameter. This model scored the same 11,765 eSNPs across 4,137 eGenes this time with a vector of 16 scores, each for each cell type. We used a label *z*-score threshold of 2 for high specificity and 1 for low specificity. To determine if each eSNP overlapped with a cell type-specific peak or enhancer, we downloaded annotated regions from Ziffra et al. [19].

We embedded the length 31 label score vectors for each eQTL into two-dimensional space using the UMAP method (as implemented in the uwot package version 0.1.10 in R) with default parameters [32]. For each gene, we considered it to have divergent eQTLs if at least two eSNPs mapped to that eGene which were not ancestors of each other in the original hierarchy, had a Euclidean distance of at least 40 between their length 31 cell type vectors (to ensure they are not highly correlated), and had a Euclidean distance of at least 8 in UMAP space (to ensure the eQTLs do not correspond to similar cell types).

### Functional validation

scRNA-ATAC multiome data was downloaded from Trevino et al. [16]. We calculated the entropy of each gene from the scRNA-seq portion of the data as described in Kannan et al. [33]. We assigned multiome cells to each eQTL if that eQTL’s eSNP was accessible in that cell but no other eSNP for the same gene was accessible. We then calculated the log-fold change in mean expression and a *p*-value (using a two tailed Wilcoxon test) for the gene for each eQTL between cells assigned to that eQTL and those not assigned to any eQTL. A false discovery rate was calculated for the *p*-values using the Benjamini–Hochberg procedure. We filtered for highly expressed genes as those that had at least 100 reads. Trevino et al. assign a cell type (not necessarily the same as the cell types used in our approach) to each cell in the multiome data based on the scRNA-seq portion of data. We calculated cell type enrichment by taking the multiome cell type assigned to each cell and for each eQTL and computing the fraction of cells assigned to that eQTL that are of that cell type over the fraction of cells not assigned to that eQTL that are that cell type.

Transcription factor (TF) binding motif disruption for each eSNP was calculated using motifbreakR (version 2.14.2) with default parameters [34]. A TF was considered expressed in a cell type if it had at least 100 reads per million mapped reads in that cell type in the Polioudakis et al. scRNA-seq data [15]. The TF by gene heatmap and TF by TF heatmap were generated using the *heatmap.2* function in R with the “symm” variable set to TRUE. The first is trimmed to only show counts greater than five and the second to only show counts greater than two. Expression UMAPs for *FABP7* and *ICA1L* were generated from CoDEx Viewer [15].

To validate the *FABP7* locus, known enhancer data was downloaded from FANTOM5 [35], and candidate cell type-specific regulatory elements were downloaded from Deng et al. [17] Massively Parallel Reporter Assay (MPRA) data for cerebral organoid and primary fetal cortical cells were also downloaded from Deng et al. The machine learning model from that paper was run on each eSNP to predict an RNA/DNA ratio for each reference and alternative allele.

## Supplementary Information

Additional file 1: Figs. S1-S13. This file contains Fig. S1. Mapping non-hierarchical cell types to eQTLs. Fig. S2. Label scores for cell types in the developing brain can be highly correlated. Fig. S3. eQTL mapped cell types overlap with cell type-specific peaks and enhancers. Fig. S4. Hierarchical annotation of cell types increases the number of detectable genes with cell type-divergent eQTLs. Fig. S5. Divergent non-hierarchical cell types for eQTLs across genes. Fig. S6. Entropy increases with the number of cell type-specific eQTLs. Fig. S7. Genes with cell type-divergent eQTLs are differentially expressed. Fig. S8. Multiome expression data for eQTLs. Fig. S9. Multiome cell type enrichment for hierarchical cell types. Fig. S10. Multiome cell type enrichment for eQTLs. Fig. S11. cell type-specific TF Binding. Fig. S12. Co-occurring TFs. Fig. S13. The *FABP7* Locus.

Additional file 2: Table S1. Full names of cell types, including both specific cell types as well as higher level annotations.

Additional file 3: Table S2. Full list of cell type annotations for eQTLs.

Additional file 4: Review history.

## Data Availability

No datasets were generated during the current study. All analyzed data is publicly available or available by request from the corresponding publications as follows: Ziffra et al. multi-sample mid-gestation human telencephalon scATAC-seq data is available from synapse.org id syn21392931 (https://doi.org/10.7303/syn21392931) [19], Wen et al. fine-mapped eQTLs are available from synapse.org id syn50897018.5 (https://doi.org/10.7303/syn50897018.5) [19], Trevino et al. scRNA-ATAC multiome data is available from GEO accession number GSE162170 [16], Deng et al. and Massively Parallel Reporter Assay (MPRA) data for cerebral organoid and primary fetal cortical cells is available from synapse.org id syn4921369 (https://doi.org/10.7303/syn4921369) [17]. Source code used in the manuscript is available under the GNU GPL-2.0 License at https://github.com/PFPrzytycki/Hierarchical_eQTL (DOI: https://zenodo.org/doi/10.5281/zenodo.11285605) [36].
