# Supplementary material for: Hierarchical annotation of eQTLs by H-eQTL enables identification of genes with cell type-divergent regulation
Source: Genome Biol. 2024 Nov 25;25:299. doi: 10.1186/s13059-024-03440-2 (PMC11587609; doi:10.1186/s13059-024-03440-2)
Supplement: Supplementary file 4 — Additional file 4: Review history. [file 13059_2024_3440_MOESM4_ESM.docx]

**1^st^ round**

**Reviewer 1**

In this paper, the authors present a network-based model for hierarchical annotation of bulk-derived eQTLs to levels of a cell type tree using single cell chromatin accessibility data and no clustering of cells into discrete cell types is needed.

I think one key limitation of the method is that it restricts to only bulk-derived eQTLs. Because it is possible a SNV is an eQTL for one or more cell types, but not at the bulk level due to heterogeneity of the cell types. If this is the case, the authors might consider some discussion of this point.

A prerequisite of using the method is that the cell type hierarchy of the tissue is known a priori. The authors state that this is taken as prior knowledge. I am concerned how easy/difficult it is to acquire such a hierarchy with cell type labels. I suggest the authors provide some advice, and better yet, detailed instruction on how to construct such a hierarchy.

The acronyms of cell types are important for this study. I am unable to find such a dictionary. A summary of the cell types and their abbreviations will be very helpful.

Out of 161,929 fine-mapped eQTLs, only 11,765 are scored because they "could be scored based on the scATAC-seq data". What are the criteria? Please elaborate.

Fig 1b. At the bottom of the hierarchy, it shows that the parent node of leave nodes usually has more eQTLs then their children. This is understandable. But why those parent nodes of internal nodes have much less eQTLs? This is very puzzling.

Fig 2a. I would suggest the authors to order the cell types by the number of eQTLs, unless the authors have more compelling reasons to keep the current order.

Fig 2b. For all the bars, does the list cover all nodes (including head, leaf and all internal)? If not, why? For the list to the right, the list seems not include all the 16 cell types in Fig 2a. Why exclude some of the cell types such as Per?
Fig 2c. Why some of the eQTLs with the same color (from the same cell type) do not cluster together, and some of the eQTLs cluster together belong to different cell types (colors)?

Fig 3b. I think genes referred here are so called "eGenes", right? I would suggest the authors to order the cell types by the number of eGenes, unless the authors have more compelling reasons to keep the current order.

Fig 4a. For the 2 genes, suggest using different color schemes with different color since their expression levels range differently.

Fig 4b. What are "multimodal cell types"? These cell types seem to be very different from the 16 cell types used earlier. This is very confusing.

Line 16 on page not-known, "… scores, each for each cell type. We used Used a label score threshold of two for high specificity" please edit.

**Reviewer 2**

The paper by Pawel F Przytycki and Katherine S Pollard applied their CellWalkR method to map eQTLs obtained from bulk brain tissues to developmental neural cell types by hierarchical annotation. It is a highly innovative approach. It integrated both single-cell ATAC-seq and scRNA-seq data for mapping the cell types. Furthermore, they dissected the expression regulation of two example genes with MPRA data to demonstrate the validity of their findings. The study targets obvious challenges in the field and provides some intriguing results. However, some additional analyses and data may help to address some concerns and confusions:
1. The authors labeled 5,889 eQTLs to hierarchical cell types with "high-specificity" with z-score > 2. Later, they called it "label score" but did not explain how this score is calculated or how it can indicate specificity. This is a critical parameter to explain to the readers. Without that, I am not convinced about the specificity.
2. Another concern is about the accuracy of cell classification. Even though they "observe that generally the scRNA-seq-based cell type annotations are enriched in the predicted cell type when the corresponding eQTL variant is accessible," the hierarchical and non-hierarchical eQTLs have clear discrepancies according to data like Fig 2.b. This suggests a potential reproducibility issue.
Can we make a list of evidence to prove that the cell types captured by CellWalkR are truly what they are? I feel that they might have chosen a difficult job given the high similarity among cell types in the fetal brain. If they could use adult brain data to prove the validity of this method, I would have more confidence about it. Brain cell type and related eQTL may have more solid data for comparison.
The high similarity among cell types in the fetal brain makes cell classification difficult. Proving the cell identity is equally difficult. Non-neuronal cells can be separated from neuronal cells easily. After that, whatever is annotated may be hard to prove.
3. "eQTL variants tend to cluster by cell type, rather than by the gene each variant is linked to." This is another interesting observation. But it is better to have some statistics to show the significance of the difference.
4. It would be nice to provide the complete list of eQTLs along with their annotated cell types using both hierarchical and non-hierarchical methods. People may have a chance to test their validity in the future or use them for other studies.
5. It is expected that the eQTL cell type classification should match up with their cell type gene expression patterns. The tree structure should indicate what eQTLs could be shared by what closely related cell types but not by the others. Such pattern should match their expression cell type specificity. Can we test that?
6. Why do internal nodes exist in some places but not others? I would expect that many eQTLs, like neurons or excitatory neurons, should map to the "internal node" that matches up a "superclass" of cell types.
7. The paper mentioned: "low overlap between regions tested in MPRAs and eQTLs" but did not provide the exact information. What are the numbers here? Is that overlap significant?
8. The MPRA validation sounds good, but no specific cell type was mentioned. The data seems to validate the eQTL in general but could not prove their cell-type specificity, right?
9. "We expanded our analysis using a machine learning model trained on MPRA data." It might be good to provide a few more words about the model and how RNA/DNA ratios can be predicted to prove the eQTLs. Again, I didn't see a place for cell-type-specificity.

In general, I like that this paper is trying to address a challenging problem and generate some potentially useful results. However, the claimed cell-type-specificity needs to be better validated.

**Reviewer 3**

Przytycki et al present a novel model that combines bulk eQTL data with single-cell chromatin accessibility (sc-ATAC-seq) data to map bulk eQTLs to a cell type hierarchy. The novelty of the paper is to treat cell types as an hierarchical graph structure instead of discrete labels. This helps the model to better capture eQTLs that are active in multiple related cell types. The model broadly agrees with our current understanding of cell-type-specific eQTL effects, which are likely to be driven by combinations of cell-type-specific transcription factors, many of which are share by related cell types. However the model makes two key assumptions which I think are worth discussing in a bit more detail in the manuscript: 1. to model assumes that the underlying causal eQTL variant (or at least the causal regulatory element) is known; 2. the model assumes that cell-type-specific chromatin accessibility is perfectly correlated with cell-type-specific gene expression.

1. Known causal variant assumption. On p 9, line 56 the authors report that they started from '161,929 fine-mapped eQTLs' from the the Wen et al paper and reduce this to 5,889 eQTLs that overlapped sc-ATAC-seq data. However, is is not cleat what the '161,929 fine-mapped eQTLs' exactly refer to. I assume that this refers to all variants that belonged to at least one SuSiE credible set from Wen et al? The authors should clarify this in their main text or Methods. If my assumption is correct, then only a very small fraction of these fine mapped eQTLs correspond to true causal variants. For example, Wen et al report that only 2,423 credible sets contained exactly one SNP and a median credible set contained five. Furthermore, fine-mapping posterior probabilities can often be mis-calibrated, which reduces the proportion fo true causal variants even further. On the other hand, eQTL causal variants are enriched in open chromatin, so overlapping credible set variants with open chromatin regions is not totally unreasonable. However, I would like to see a bit more discussion on what proportion of the reported 5,889 eQTLs are likely to correspond to true causal variants and how uncertainty in the ascertainment of causal variants affects the model results.
2. The model assumes that cell-type-specific chromatin accessibility is perfectly correlated with cell-type-specific gene expression. While this likely to often be the case, we also know that it is not always the case. For example, Alasoo et al 2018 (https://doi.org/10.1038/s41588-018-0046-7) reported several examples of 'enhancer priming', where genetic effects on chromatin accessibility were visible already in naive cells but effect on gene expression only appeared after immune stimulation. Similar effects have also been reported in neural progenitors (https://www.biorxiv.org/content/10.1101/2023.02.07.527357v2). It is possible that similar effects could take place in cell type hierarchies where chromatin becomes accessible in a cell type higher in the hierarchy, but the effect on gene expression manifests only in a subset of the cell types in lower levels of the hierarchy. I think it is fair to state that we do not currently have an accurate estimate of how common enhancer priming in cell type hierarchies is, but I think it would be helpful to discuss this in the main text.

**Authors’ response**

**Reviewer #1**

1. **I think one key limitation of the method is that it restricts to only bulk-derived eQTLs. Because it is possible a SNV is an eQTL for one or more cell types, but not at the bulk level due to heterogeneity of the cell types. If this is the case, the authors might consider some discussion of this point.**

We completely agree with this observation about the limitations of working with bulk-derived eQTLs and have expanded our discussion section to address this point. While the goal of the method developed in our manuscript is to annotate such eQTLs, we expect that many cell type specific eQTLs are simply missed due to low prevalence in bulk samples. Our method could also be applied to cell type specific eQTLs to annotate them using cell type labels from an external source or to get a probabilistic mapping for labels from the same dataset.

2. **A prerequisite of using the method is that the cell type hierarchy of the tissue is known a priori. The authors state that this is taken as prior knowledge. I am concerned how easy/difficult it is to acquire such a hierarchy with cell type labels. I suggest the authors provide some advice, and better yet, detailed instruction on how to construct such a hierarchy.**

There is indeed an enormous variety in the availability and quality of cell type hierarchies. Detailed hierarchies are the results of many years of research and have only recently started to become more abundant. Examples include a cell-type tree of prenatal mouse development [Qiu et al. 2024], C. elegans embryogenesis [Packer et al. 2019], hematopoiesis [Weinreb et al. 2020], and D. melanogaster embryogenesis [Calderon et al. 2022]. On the other end, basic hierarchies can be generated directly from scRNA-seq data with computational methods such as treeArches [Michielsen et al. 2023] and CellHint [Xu et. al 2023]. Notably, our proposed method also works with partial hierarchies in cases where there is a limited amount of prior knowledge. We have expanded our discussion section to comment on the availability of cell type hierarchies.

3. **The acronyms of cell types are important for this study. I am unable to find such a dictionary. A summary of the cell types and their abbreviations will be very helpful.**

We have added a summary of cell type abbreviations, which now appears in Supplementary Table 1.

4. **Out of 161,929 fine-mapped eQTLs, only 11,765 are scored because they "could be scored based on the scATAC-seq data". What are the criteria? Please elaborate.**

Our model is only able to score eQTLs that overlap at least one read from at least one cell in the scATAC-seq data. Those that have no overlap could not be scored. We have updated the text to explicitly state this overlap requirement.

5. **Fig 1b. At the bottom of the hierarchy, it shows that the parent node of leave nodes usually has more eQTLs then their children. This is understandable. But why those parent nodes of internal nodes have much less eQTLs? This is very puzzling.**

This is a great observation and is due to technical details in the method. For any significant parent node, if both child nodes are also significant, only the parent label is kept. This is often the case for the parent to two highly correlated leaf nodes, but is rarely the case higher up the tree where the nodes are not as correlated. We believe this best reflects the underlying biology, and have added to the text to emphasize these details and their effects.

6. **Fig 2a. I would suggest the authors to order the cell types by the number of eQTLs, unless the authors have more compelling reasons to keep the current order.**

For consistency, we opted to use the order of cell types from the original cell tree for every figure throughout the manuscript (shown at the bottom of Figure 2). This enables easy comparisons across figure panels. We prefer this ordering but are open to changing the ordering on a per-figure basis if this would improve overall readability.

7. **Fig 2b. For all the bars, does the list cover all nodes (including head, leaf and all internal)? If not, why? For the list to the right, the list seems not include all the 16 cell types in Fig 2a. Why exclude some of the cell types such as Per?**

Figure 2b is showing every cell type with a non-zero count. Cell types that appear as non-zero counts in Figure 2a but are not shown in Figure 2b are due to the package we used plotting a small bar at 0 when the count is equal to 0. Thanks for pointing out that this is confusing. We agree and have fixed the plot to make 0 counts clear by removing the small bar.

8. **Fig 2c. Why some of the eQTLs with the same color (from the same cell type) do not cluster together, and some of the eQTLs cluster together belong to different cell types (colors)?**

This is a very interesting observation and is likely due to the continuous nature of cell states during brain development. To investigate this, we took a closer look at some of the eQTLs that were labeled as newborn excitatory neurons (ExN) but split across multiple clusters and indeed found a gradient of neuron maturity (ExM score) as shown in the figure below. The continuity of cell states is a very exciting topic and we have now noted it in our discussion.

1. **Fig 3b. I think genes referred here are so called "eGenes", right? I would suggest the authors to order the cell types by the number of eGenes, unless the authors have more compelling reasons to keep the current order.**

Yes, these are indeed eGenes. To address this comment, we have updated the manuscript throughout to refer to them as eGenes where appropriate. As with the ordering of cell types described above, these also follow the same ordering from the original cell type tree.

2. **Fig 4a. For the 2 genes, suggest using different color schemes with different color since their expression levels range differently.**

While this is a good suggestion, we are unfortunately unable to control the colors as the figures are generated by a server that hosts this particular data (CoDEx Viewer).

3. **Fig 4b. What are "multimodal cell types"? These cell types seem to be very different from the 16 cell types used earlier. This is very confusing.**

That is correct, the “multimodal cell types” are a set of non-hierarchical cell type labels from Trevino et al. used as an external second validation dataset, while our hierarchy of cell types is derived from the labels gendered by Polioudakis et al. We attempted to distinguish the two sets of labels by calling the former “multiome cell types” but this comment highlights the fact that this was insufficient. To address this, we have now updated the text to clarify the distinction between these two sets of cell type labels and their sources.

4. **Line 16 on page not-known, "… scores, each for each cell type. We used Used a label score threshold of two for high specificity" please edit.**

Thank you, this typo has been fixed.

**Reviewer #2**

1. **The authors labeled 5,889 eQTLs to hierarchical cell types with "high-specificity" with z-score > 2. Later, they called it "label score" but did not explain how this score is calculated or how it can indicate specificity. This is a critical parameter to explain to the readers. Without that, I am not convinced about the specificity.**

We apologize for any confusion with our terminology. The label score is just our name for the z-score for each label and a threshold of 2 is used throughout the paper with the lone exception being the examination of “low-specificity” labels, where we used a threshold of 1. We have updated the text to clarify this.

2. **Another concern is about the accuracy of cell classification. Even though they "observe that generally the scRNA-seq-based cell type annotations are enriched in the predicted cell type when the corresponding eQTL variant is accessible," the hierarchical and non-hierarchical eQTLs have clear discrepancies according to data like Fig 2.b. This suggests a potential reproducibility issue.  Can we make a list of evidence to prove that the cell types captured by CellWalkR are truly what they are? I feel that they might have chosen a difficult job given the high similarity among cell types in the fetal brain. If they could use adult brain data to prove the validity of this method, I would have more confidence about it. Brain cell type and related eQTL may have more solid data for comparison. The high similarity among cell types in the fetal brain makes cell classification difficult. Proving the cell identity is equally difficult. Non-neuronal cells can be separated from neuronal cells easily. After that, whatever is annotated may be hard to prove.**

We selected the developing brain for this study because it has many highly correlated and difficult to disentangle cell types, making it well suited for a hierarchical analysis. While we validated the original non-hierarchical approach in our CellWalker paper [Przytycki and Pollard 2021] where we found it was better able to label cells than Seurat, especially from rare cell types, the question of robustness of the hierarchical annotations relative to the non-hierarchical annotations is very interesting. There are indeed some discrepancies visible in Figure 2b, but 81.4% of all annotations remain consistent within a level of the hierarchy (direct parents or siblings), which is now noted in our results. The most notable exception are the 96 eQTLs that switched from being labeled as maturing upper layer excitatory neurons to deep layer neurons. Taking a closer look at these, of those that overlap known enhancers, 25% overlap an enhancer annotated for both upper layer and deep layer neurons, so they may in fact be pleiotropic enhancers, which we touch on in our discussion section. Another, perhaps surprising, observation is that 8.3% of eQTLs revert to a broad label, implying that there is some overfitting to specific cell types when a hierarchy is not used.

As an additional validation, we have now run our model on adult brain data using a set of adult brain cell-type specific eQTLs from Bryos et al. [Bryos et al. 2022]. Among this set of eQTLs, we found that a striking 58% were marked as significant in the correct corresponding cell type by CellWalker when using scATAC-seq data for the adult brain taken from Corces et al. [Corces et al. 2020]. We believe this is as high a correspondence as we could hope for given that eQTLs are scRNA-seq based while our model uses scATAC-seq data.

Other lines of evidence supporting our cell type annotations come from the multimodal data from Trevino et al. [Trevino et al. 2021], which was not used in the original annotations. In this data, we observe that in cells where the given eQTL is accessible, the corresponding transcriptome based cell type label matches are strongly enriched for the label assigned by CellWalker to the eQTL as summarized in the table below:

| **Multimodal Cell Type** | **Hierarchical Cell Type** | **Median Enrichment** |
| --- | --- | --- |
| Cycling Progenitors | oRG/vRG/OPC/PpG2M/PgS | 2.27 |
| Endothelial Cells | Endothelial Cells | 63.6 |
| GluN2 | ExM/ExN | 1.89 |
| GluN3 | ExM/ExN | 1.09 |
| GluN4 | ExM/ExN/IP/ExM-U | 4.54 |
| GluN5 | ExM/ExN/IP/ExM-U | 2.36 |
| IN1 | InMGE/InCGE | 1.5 |
| IN2 | InMGE/InCGE | 1.69 |
| IN3 | InMGE/InCGE | 2.8 |
| mGPC/OPC | oRG/vRG/OPC | 4.25 |
| nIPC/GluN1 | ExM/ExN/IP | 1.65 |
| RG | oRG/vRG | 3.28 |
| Subplate Neurons | ExDp2/ExDp1 | 2.11 |

Finally, when looking at the subset of eQTLs that correspond to cell-type divergent regulation (e.g. a gene has two or more eQTLs assigned to different cell types), we found that each individual eQTL for the same gene, when accessible in a cell, corresponds to a higher chance of that cell being annotated to the corresponding cell type. The plots below show three of the most common combinations of divergent regulation.

1. **"eQTL variants tend to cluster by cell type, rather than by the gene each variant is linked to." This is another interesting observation. But it is better to have some statistics to show the significance of the difference.**

We apologize for the omission of the underlying statistics for this observation. We have now quantified the the distances between pairs of eQTLs either assigned to the same gene or to the same hierarchical cell type, and confirmed that the observation is true both in high-dimensional space and in the embedded UMAP space (*p*-value <  0.05 in both cases with a two-tailed Wilcoxon rank-sum test). We have updated the text to add these statistics.

1. **It would be nice to provide the complete list of eQTLs along with their annotated cell types using both hierarchical and non-hierarchical methods. People may have a chance to test their validity in the future or use them for other studies.**

We have added a table of annotated eQTLs that includes both sets of annotations for future usage (now appear in Supplementary Table 2).

2. **It is expected that the eQTL cell type classification should match up with their cell type gene expression patterns. The tree structure should indicate what eQTLs could be shared by what closely related cell types but not by the others. Such pattern should match their expression cell type specificity. Can we test that?**

While it should be possible to test how genes are expressed across a cell type hierarchy, it is key to note that the cell type annotation is for the eQTL variant rather than the eGene, and therefore eQTL cell type classification should only partially match cell type gene expression patterns, as t each eQTL may corresponds to the expression of the gene in that particular cell type. One way to test for this would be to use multiome (scRNA + scATAC) data and check the accessibility of each eQTL against the expression of its eGene in that same cell. Unfortunately, however, existing multiome data is too sparse to conduct these tests.

3. **Why do internal nodes exist in some places but not others? I would expect that many eQTLs, like neurons or excitatory neurons, should map to the "internal node" that matches up a "superclass" of cell types.**

While the internal node always exists, in many cases very few or no eQTLs map to it. We agree that in some cases it is a bit surprising to see so few eQTLs map to “superclasses".” For example, only 2 eQTLs were broadly neuronal, which may be due to that class containing both excitatory and inhibitory neurons. While there was no class for all excitatory neurons because in the hierarchy deep layer neurons were split from other excitatory neurons, the most similar nodes to such a description, ExM/ExN, ExM/ExN/IP and  ExM/ExN/IP/ExM-U had 505 (8.6% of all mapped eQTLs), 166 (2.8%), and 3 (<0.1%) eQTLs map to them respectively. We interpret this to imply that upper layer excitatory neurons in particular have very different regulatory elements compared to the newborn and maturing excitatory neuron classes.

4. **The paper mentioned: "low overlap between regions tested in MPRAs and eQTLs" but did not provide the exact information. What are the numbers here? Is that overlap significant?**

While overall 1.8% of the full set of all fine-mapped eQTLs mapped to regions tested in MPRAs, a full 31.9% of those involved in divergent regulation did, which is a significant enrichment in overlap. However, one major advantage of the  machine learning model is that it can score both the reference and variant allele for each eQTL therefore providing an estimate of the effect of the specific eQTL, rather than just establishing that the eQTL lies in a functional region. We have clarified the text to indicate this specific advantage of the machine learning model’s predictions.

5. **The MPRA validation sounds good, but no specific cell type was mentioned. The data seems to validate the eQTL in general but could not prove their cell-type specificity, right?**

It is unfortunately true that current MPRA protocols are not able to capture cell-type specificity in the brain. Single-cell MPRAs are currently in development, but primary successes have been in cell lines, with scaling to contexts beyond that is still ongoing research. We have updated our discussion section to mention single-cell MPRAs and the potential use of CRISPRi/a plus gene expression in sorted cell types as possible future directions.

6. **"We expanded our analysis using a machine learning model trained on MPRA data." It might be good to provide a few more words about the model and how RNA/DNA ratios can be predicted to prove the eQTLs. Again, I didn't see a place for cell-type-specificity.**

Indeed, there is unfortunately no cell type specificity in these predictions as the model was trained on bulk MPRA studies. Our primary goal of using the model was to show that there are multiple functional eQTLs for the same gene in the developing brain, even if we can’t see if they are cell-type specific. We have updated the manuscript to both give a more detailed description of the model and to mention its shortcomings.

**Reviewer #3**

1. **The model assumes that the underlying causal eQTL variant (or at least the causal regulatory element) is known. On p 9, line 56 the authors report that they started from '161,929 fine-mapped eQTLs' from the the Wen et al paper and reduce this to 5,889 eQTLs that overlapped sc-ATAC-seq data. However, it is not clear what the  '161,929 fine-mapped eQTLs' exactly refer to. I assume that this refers to all variants that belonged to at least one SuSiE credible set from Wen et al? The authors should clarify this in their main text or Methods.  If my assumption is correct, then only a very small fraction of these fine mapped eQTLs correspond to true causal variants. For example, Wen et al report that only 2,423 credible sets contained exactly one SNP and a median credible set contained five. Furthermore, fine-mapping posterior probabilities can often be mis-calibrated, which reduces the proportion of true causal variants even further. On the other hand, eQTL causal variants are enriched in open chromatin, so overlapping credible set variants with open chromatin regions is not totally unreasonable. However, I would like to see a bit more discussion on what proportion of the reported 5,889 eQTLs are likely to correspond to true causal variants and how uncertainty in the ascertainment of causal variants affects the model results.**

This brings up a number of very interesting discussion points. It is absolutely true that eQTLs are not necessarily causal. While we are able to map eQTLs to cell types, the question of causality remains open. The main evidence we have for a subset of our mapped eQTLs being causal comes from overlapping them with MPRAs and testing the variants with the MPRA-based machine learning model. As mentioned in the response to reviewer #2, while overall 1.8% of the full set of all fine-mapped eQTLs (which indeed refers to all variants that belonged to at least one SuSiE credible set from Wen et al.) mapped to regions tested in MPRAs, a full 31.9% of those involved in divergent regulation did, which is a significant enrichment in overlap, as would be expected from overlapping open chromatin. Testing the reference and alternative alleles of those eQTLs with the machine learning model suggested that many of them are at least functional, if not causal (Figure 4e). 23% are predicted to have a log-fold Alt/Ref RNA/DNA ratio of at least 0.2, which is considered to be functional. CRISPR experiments and MPRAs are ways to get at that in future work using our results to help design the experiments. We have expanded our results and discussion section for clarity on causal eQTLs.

2. **The model assumes that cell-type-specific chromatin accessibility is perfectly correlated with cell-type-specific gene expression. While this is likely to often be the case, we also know that it is not always the case. For example, Alasoo et al 2018 (https://doi.org/10.1038/s41588-018-0046-7) reported several examples of 'enhancer priming',  where genetic effects on chromatin accessibility were visible already in naive cells but effect on gene expression only appeared after immune stimulation. Similar effects have also been reported in neural progenitors (https://www.biorxiv.org/content/10.1101/2023.02.07.527357v2). It is possible that similar effects could take place in cell type hierarchies where chromatin becomes accessible in a cell type higher in the hierarchy, but the effect on gene expression manifests only in a subset of the cell types in lower levels of the hierarchy. I think it is fair to state that we do not currently have an accurate estimate of how common enhancer priming in cell type hierarchies is, but I think it would be helpful to discuss this in the main text.**

We completely agree that there are many important caveats to linking cell-type-specific chromatin accessibility to cell-type-specific gene expression. We have expanded the text to touch on some of these topics including the possibility of enhancer priming. In particular, the notion that such an effect could take place in cell type hierarchies with accessibility and gene expression manifesting on different levels of the hierarchy is particularly intriguing and we have added it to our discussion as well.

**2^nd^ round**

**Reviewer 1**

I do not have additional comment.

**Reviewer 2**

The revision clarified many things and answered most of my previous questions. However, I still have problems understanding some of the issues. I am sorry for not understanding the methodology well.
As I understand, the extended CellWalkR connects a hierarchical tree (based on prior knowledge of cell lineage, with cell type label) to a cell network (based on the similarity of scATAC-seq results). I assume the “random walk” starts from the nodes in the cell network, not in the hierarchical tree. Right? Will we walk between all possible cell nodes in the cell network? Does the walk require passing through the tree? How is the information flow calculated? What is the probability? How is the probability calculated? I could not find explanations in the manuscript or the original CellWalkR paper.
“given a set of bulk-derived eQTLs, each eQTL is mapped to hierarchical cell types using
the calculated flow of information and the chromatin accessibility of individual cells.” This statement is so vague. How could the flow of information and chromatin accessibility of individual cells help map eQTL to the hierarchical cell type? I don’t understand.
If I understand it correctly, CellWalkR’s job is to relate the hierarchical tree to the scATAC-seq cells. How eQTL is mapped to the hierarchical cell type is not clear. Is it purely by overlapping eSNPs to ATAC-seq reads? But the authors made it sound more complex than that. Where is the flow of information used?
“Overlapping with one read from at least one cell in the scATAC-seq data” is a questionable criterion to me. The authors talked about overlap between eQTL and ATAC-seq read; it must be eSNPs overlap with ATAC-seq reads. ATAC-seq data is quite noisy. How do we know that one read is biologically meaningful, relating to regulatory elements related to cell-type specific gene expression? I would consider only the peaks that have established association with gene expression useful to guide cell classification.
One of the major drawbacks of the method is that only a tiny proportion of eQTL could be mapped to a cell type tree. I am curious why not use scRNA-seq data in the CellWalkR model? Will that improve the mapping rate? In the original CellWalkR paper, the authors had “Single-cell RNA sequencing (scRNA-seq) is less sparse than scATAC-seq and hence can identify dozens of cell types in complex tissues, but it does not pinpoint regulatory elements.” I am not sure if that is the justification. Can we count on each read of ATAC-seq to pinpoint regulatory elements? I am afraid not. Cell-type-specificity of gene expression might be more solid, or at least similarly solid, than ATAC-seq peaks.
The purpose of this study is to assign bulk tissue eQTL to a specific cell type tree or lineage. Unfortunately, MPRA experiments and the associated machine-learning model do not have cell-type specificity. Therefore, they deviate from the core topic. This should be acknowledged.
“eQTL” should be strictly used to describe the SNP-gene paired relationship. “eSNP” and “eGene” should be used to describe the SNP and gene involved. The paper mixed up eSNP with eQTL, and used eQTL to describe eSNP in many places.

**Reviewer 3**

The authors have acceptably addressed all of my concerns.

**Authors’ response**

Reviewer #2:

1)As I understand, the extended CellWalkR connects a hierarchical tree (based on prior knowledge of cell lineage, with cell type label) to a cell network (based on the similarity of scATAC-seq results). I assume the “random walk” starts from the nodes in the cell network, not in the hierarchical tree. Right? Will we walk between all possible cell nodes in the cell network? Does the walk require passing through the tree? How is the information flow calculated? What is the probability? How is the probability calculated? I could not find explanations in the manuscript or the original CellWalkR paper.

We apologize for the lack of clarity and details in describing how information is propagated in our network. The original model is described in detail in the 2021 Przytycki and Pollard paper “CellWalker integrates single-cell and bulk data to resolve regulatory elements across cell types in complex tissues” while the CellWalkR paper is an application note describing software that we extended for this manuscript. In this work, a walk can start on any cell or label, including internal nodes of the tree, and similarly can end on any cell, label, or internal node. Information flow is calculated as the steady state of random walk with restarts and the output is the stochastic steady-state transition matrix (“influence matrix”), which gives the probability that a walk starting at each node in the graph will end at each other node in the graph (including internal nodes of the hierarchy). Finally, while there is no requirement that any given walk pass through the hierarchical tree, one can focus an analysis on this (or any other) subset of entries in the influence matrix. For the eQTL analyses in this manuscript, we only use the probabilities for walks started on cells and ended at portions of the tree.

We thank the reviewer for suggesting that we add more methodological detail to the manuscript. To address this, we updated the text of our manuscript to include the above details as well as the exact formulation of the random walk problem and how the solution is calculated.

2)“given a set of bulk-derived eQTLs, each eQTL is mapped to hierarchical cell types using the calculated flow of information and the chromatin accessibility of individual cells.” This statement is so vague. How could the flow of information and chromatin accessibility of individual cells help map eQTL to the hierarchical cell type? I don’t understand.

We appreciate the reviewer pointing out that our explanation was vague, and we are glad to clarify. After the stochastic steady-state transition matrix is calculated, as described above, the chromatin accessibility of individual cells is overlapped with the locations of eSNPs to calculate the probability that a walk from each cell where the eSNP is accessible (i.e. there is at least one scATAC-seq read overlapping that eSNP in that cell) ends at each node of the tree. The probabilities are then summed across all the cells from which walks started. We revised this part of the text to provide these important details.

3)If I understand it correctly, CellWalkR’s job is to relate the hierarchical tree to the scATAC-seq cells. How eQTL is mapped to the hierarchical cell type is not clear. Is it purely by overlapping eSNPs to ATAC-seq reads? But the authors made it sound more complex than that. Where is the flow of information used?

We are happy to explain this better. In our method, the overlap is used to select which cells should be used, after which point the random walks are used to assign hierarchical cell types to the eSNPs. In other words, the cell type labeling method uses genome-wide chromatin accessibility of cells, and for a given eSNP, the label is determined using the distribution of labels for all cells in which the eSNP is accessible. The text has been updated to include this more detailed explanation.

We also compared our hierarchical labels to overlapping eSNPs directly with scATAC- seq peaks (Supplementary Figure 3), but we note that such an overlap approach does not assign hierarchical labels.

4)“Overlapping with one read from at least one cell in the scATAC-seq data” is a questionable criterion to me. The authors talked about overlap between eQTL and ATAC-seq read; it must be eSNPs overlap with ATAC-seq reads. ATAC-seq data is quite noisy. How do we know that one read is biologically meaningful, relating to regulatory elements related to cell-type specific gene expression? I would consider only the peaks that have established association with gene expression useful to guide cell classification.

Thanks for this question. We agree that accessibility of a nucleotide is difficult to measure in individual cells using scATAC-seq. This is why we use inferences (based on genome-wide accessibility) from all cells with any evidence of accessibility at a nucleotide of interest to make inferences for that nucleotide, as described above. Indeed, one of the main problems that CellWalker is designed to overcome is that scATAC-seq data is very sparse and noisy.

The network that is constructed by CellWalker features both cell-to-cell and label-to-cell edges. We are grateful for the opportunity to explain here and in the revised text how both types of edges are defined for the eQTL use case that we explore in this manuscript. The cell-to-cell edges are intended to help with sparsity and noise issues by identifying similar cells to share information with (based on genome-wide accessibility), in a sense smoothing over the noise inherent in the data from individual nucleotides in individual cells. The label-to-cell edges consider marker gene accessibility for defined cell types. Using multiple genes, known to have differences in expression and accessibility between cell types, is our approach to addressing effects of scATAC-seq noise on label-to-cell edges. Established links between regulatory elements and gene expression could be another option for these edges but this set of edges is likely to be much sparser than the set of edges we use.

5)One of the major drawbacks of the method is that only a tiny proportion of eQTL could be mapped to a cell type tree. I am curious why not use scRNA-seq data in the CellWalkR model? Will that improve the mapping rate? In the original CellWalkR paper, the authors had “Single-cell RNA sequencing (scRNA-seq) is less sparse than scATAC-seq and hence can identify dozens of cell types in complex tissues, but it does not pinpoint regulatory elements.” I am not sure if that is the justification. Can we count on each read of ATAC-seq to pinpoint regulatory elements? I am afraid not. Cell-type- specificity of gene expression might be more solid, or at least similarly solid, than ATAC-seq peaks.

scATAC-seq and scRNA-seq each provide unique opportunities for understanding cell- type specific gene regulation. In our case, scATAC-seq is suited for studying eSNP specificity in relation to the corresponding eGene. In particular, we can predict cell-type information for the eSNP rather than the eGene. However, in our model, scRNA-seq is actually used to define the labels, so both types of information are being considered, though not for individual cells in the case of scRNA-seq data. When available, multiome data could be used to consider expression and accessibility in individual cells at the same time (multiome data would be necessary to link scRNA-seq to non-coding regions), which is a new direction we are actively exploring.

6)The purpose of this study is to assign bulk tissue eQTL to a specific cell type tree or lineage. Unfortunately, MPRA experiments and the associated machine-learning model do not have cell-type specificity. Therefore, they deviate from the core topic. This should be acknowledged.

Yes, it is unfortunately true that the MPRA experiments and the associated machine learning model do not have cell-type specificity, and we now acknowledge this in the text.

7)“eQTL” should be strictly used to describe the SNP-gene paired relationship. “eSNP” and “eGene” should be used to describe the SNP and gene involved. The paper mixed up eSNP with eQTL, and used eQTL to describe eSNP in many places.

Thank you for pointing this out, we have updated the text to correctly label eSNPs where appropriate. We now employ the terminology “eSNP” for variants, “eGene” for genes, and “eQTL” for variant-gene pairs.

**3^rd^ round**

**Reviewer 1**

In this revision, the authors addressed 7 comments raised by a reviewer (not me). I felt that the authors did a fine job addressing them and modified their manuscript accordingly. Most of the questions raised are fair. It is helpful to clarify the details of the method. It is also helpful to acknowledge the limitations of the method because single cell RNA-seq and ATAC-seq data are noisy and sparse. The authors are working on a difficult problem, and they did their best to cope with the caveats of the single cell data. And I would say their proposed method indeed has several advantages over existing methods. I don't fully agree with comment 7 of the reviewer. I think it is most people's understanding that eQTL means the variant or SNP, not the relationship, eSNP is not commonly used. But this is not a big deal.

**Reviewer 2**

No further comment.
